# Analysis of the chain mediation effect between intergenerational support and mental health of older adults in urban China: a structural equation model

**DOI:** 10.1186/s12877-024-04883-9

**Published:** 2024-04-08

**Authors:** Jiahong Xu, Youwei Wang, Justin C. Cheung, Yanlong Yin

**Affiliations:** 1 Department of Sociology, Zhou Enlai School of Government, Nankai University, Tianjin, China; 2 School of Sociology & Anthropology, Sun Yat-sen University, No. 135, Xingang West Road, Haizhu District, Guangzhou, Guangdong Province China; 3 Wee Kim Wee School of Communication and Information, Nanyang Technological University, Singapore, Singapore; 4 Department of Social Security, School of Public Administration, Hebei University of Economics and Business, Shijiazhuang, China

**Keywords:** Intergenerational support, Mental health, Intergenerational solidarity

## Abstract

**Background:**

This study aimed investigate the impact of intergenerational support on the mental health of older adults in urban China. It also sought to evaluate the chain mediation effect of attitudes toward younger people and willingness to interact with younger people within a non-familial context between intergenerational support and mental health.

**Methods:**

Data were derived from a community survey that adopted quota sampling in mainland China in 2022 (*N* = 780). Structural equation modeling was used to analyze the data, and the bootstrap technique was used to test the mediation effect.

**Results:**

A significant positive association was found between intergenerational support and the mental health of older adults in urban China (B = 0.852, 95% confidence interval CI [0.157,1.617]). Intergenerational support had a specific indirect effect on mental health through older adults’ attitudes toward younger people within a non-familial context (B = 0.665, 95% CI [0.443,1.046]). There was a chain mediation effect (B = 0.126, 95% CI [0.069,0.224]) in relation to attitudes toward younger people and the willingness to interact with younger people between intergenerational support and mental health. Mediation accounted for 44.44% of the total effects in the model.

**Conclusion:**

These findings help identify modifiable factors that can improve the mental health of older adults. In line with the proposed serial multiple mediation model, this study provides theoretical and practical insights concerning the synergistic effect of intergenerational support at the family level and intergenerational interaction at the community level. Policy and social service implications are also discussed.

## Background

Mental well-being has emerged as an important concern in international public health and received significant attention within the framework of global development. This emphasis is underscored by its inclusion in the United Nations Sustainable Development Goals, which targets realization by 2030 [1]. Poor mental health is a risk factor for multiple diseases, is a comorbidity of many physical health problems, and complicates the care of co-occurring diseases [2]. These intersections invariably present a major challenge for the geriatric population. China, the world’s most populous country, is rapidly becoming one of the most rapidly aging countries. As of 2020, the number of people in China aged 60 years and older had reached 264.02 million, accounting for 18.7% of the total population [3]. Given this notable aging population trend, mental health has become a public health concern in China. A meta-analysis found that the psychological health of older adults in China has been declining annually [4], with approximately 36.9% of older adults reporting depressive symptoms in the most recent nationally representative survey [5]. The risks in this situation need to be highlighted. The World Health Organization (WHO) recognizes the essential role of mental health in achieving overall health [6]. Mental health, a multifaceted construct, has been delineated by the WHO as a “state of well-being in which an individual realizes his or her own abilities, can cope with the normal stresses of life, can work productively, and is able to make a contribution to his or her community” [7]. Consequently, this research endeavors to frame mental health not simply in binary terms according to the presence or absence of psychiatric disorders but rather through a three-dimensional lens: manifestations of depressive indicators, feelings of solitude, and the degree of life contentment [8].

Contemporary research has underscored the salient relationship between positive intergenerational relationships and desirable mental health outcomes. These outcomes include reduced depressive symptoms and enhanced well-being, cognitive functioning, happiness, and life content [9–13]. Research has also suggested that older adults who receive augmented support display higher psychological well-being [14]. The protective effects of intergenerational support on mental health outcomes also apply among older adults in urban China in relation to loneliness, insomnia and depression [15]. However, contrasting findings have emerged, with some studies reporting that high levels of support from adult children can be detrimental [16], or exert minimal influence on older adults’ well-being [17]. Additionally, some studies have identified inconclusive associations between intergenerational support and geriatric mental health, especially depression [18–19]. A plausible explanation for these disparate findings is the limited scope of prior research, which may not have holistically encapsulated the intricate mechanisms underlying the impact of intergenerational support on geriatric mental health. In other words, there may be some variables acting as intermediaries, given the trends toward urbanization and family nucleation such that intergenerational solidarity may develop within a non-familial context. We propose a nuanced multi-mediational model of mental health to evaluate whether intergenerational support potentially influences attitudes toward younger people and the willingness to interact with younger people within a non-familial context, thereby affecting mental health among older adults.

### Theoretical considerations

In China, adult children are expected to provide support to their parents beyond conceptions of repayment, thus adhering to traditional filial cultural expectations [20]. Such support, which is emblematic of familial belonging, concern, and care, could significantly bolster the mental well-being of older adults. Indeed, advancing age often heralds a diminution in economic income and self-care ability concomitant with the emergence of augmented needs such as medical expenditures. In this sense, intergenerational support encompassing financial, material, physical, and emotional facets could play an important role in relieving the challenges faced by older adults, thereby catalyzing a positive psychological response.

Drawing on the intergenerational solidarity model [21–22], we conceptualize intergenerational support as not only including financial and material support but also as involving instrumental and emotional dimensions. We consider that the inclusion of these two support dimensions is integral to the concept of intergenerational support, as instrumental and emotional support reflect relational closeness and quality better than financial and material support alone. Taken together, we propose the following hypothesis:

#### Hypothesis 1

Intergenerational support positively affects older adults’ mental health.

Drawing on empirical findings, we formulated a model delineating the impact of intergenerational support on geriatric mental health, incorporating attitudes toward younger generations within a non-familial context as a potential mediating factor. Contemporary sociocultural changes in living and working patterns have curtailed opportunities for intergenerational social interactions. The trend toward a nuclear familial structure and age-specific segregation has diminished the prevalence of multigenerational households and age-inclusive community settings [23–24]. Such dynamics can increase the risk of social isolation and poor mental well-being among older adults [25–26].

Empirical evidence also underscores the pivotal role of familial ties in knowledge dissemination across age groups and in fostering positive attitudes toward other age groups [27]. Affective transfer theory suggests that residual emotional arousal from one stimulus can potentiate an emotional response to a subsequent one, a process not confined to a singular emotional state [28–29]. From this perspective, we postulate that positive attitudes toward the younger generation, cultivated through intergenerational familial interaction, may generalize to include broader youth demographics in society. This shift could counteract age discrimination stemming from generational separation in the community, thereby fostering desirable mental wellbeing outcomes for older adults in modern society. Therefore, we propose the following hypothesis:

#### Hypothesis 2

Intergenerational support has a specific indirect effect on mental health through older adults’ attitudes toward younger people in a non-familial context.

In addition to older adults’ attitudes toward younger non-familial people, their willingness to interact with younger people may serve as another mediating role between intergenerational support and mental health. According to activity theory, the determinants of life satisfaction and mental well-being during the aging process are contingent upon remaining active and the ability to counteract the degradation of social networks and activities [30]. We considered it likely that older adults who are beneficiaries of intergenerational support from adult children would encounter fewer daily stressors. This, in turn, would allow them to be involved in more social activities, given their available leisure time and resources. However, some researchers have argued that negative age stereotypes may be rooted in peer relationships within an age segregation context, whereas family and kinship play important roles in providing a basis for age integration within a non-familial context [31]. In other words, intergenerational interactions within the family provide a basis for older adults to participate in intergenerational programs and activities at the community level. Synthesizing these perspectives, a potential interrelationship that encompasses intergenerational support, the willingness to participate in social activities with non-familial younger individuals, and mental health emerges. Consequently, we propose the following hypothesis:

#### Hypothesis 3

Intergenerational support has a specific indirect effect on mental health through older adults’ willingness to interact with younger people in a non-familial context.

Furthermore, we posit a serial mediating effect, wherein older adults’ attitudes toward younger people in a non-familial context facilitate a transition to their willingness to interact with these younger people, thereby influencing the relationship between intergenerational support and mental health. Factors such as the rapid development of technology, changes in family structure, relationship breakdowns within families, and migration are believed to be catalysts for generational segregation [32].

From the above studies, it is evident that attitudes toward younger people and the willingness to interact with younger people within a non-familial context are fundamentally critical factors for establishing and maintaining key relationships based on the mental well-being needs of older adults, particularly with the majority of older adults being community-based in modern society [23–26, 31]. Empirical research has underscored that attitudes stemming from direct experience promote greater attitude-behavior consistency [33–34]. This body of evidence lends credence to the notion that positive attitudes toward younger people can galvanize older adults’ willingness to interact in non-familial settings. Hence, we propose the following hypothesis:

#### Hypothesis 4

The transition from attitudes toward younger people to older adults’ willingness to interact with younger people within a non-familial context has a serial mediating effect on the relationship between intergenerational support and mental health.

## Methods

### Data collection

The data for this study were obtained from a community survey conducted in China in 2022. A quota-sampling method was used to select samples from two cities in Central and Eastern China. Five districts were randomly selected from each city. Second, two communities were randomly selected from each district, for a total of ten communities in each city. Forty respondents aged 60 years or older were selected from each community. The age and gender ratios of the respondents were controlled in accordance with their statistical representation in the latest local demographic data. The respondents had to meet the following criteria: (1) have a local household registration status, (2) be aged 60 years or older, and (3) have lived in their local community for more than 180 days in the previous 12 months.

Trained interviewers conducted face-to-face interviews at the respondents’ homes and local community centers. All the respondents signed an informed consent form before the start of the survey. They were also informed of their right to withdraw from the study at any time point. A total of 853 people were interviewed, 800 of whom completed the interviews. After removing samples with missing values for key variables, a total sample of 780 individuals was included in the analysis.

### Measurement

#### Dependent variable

The dependent variable was mental health, which is a latent variable consisting of three aspects: depressive symptoms, life satisfaction, and loneliness. Depression was measured using the Center for Epidemiologic Studies Depression Scale (CES-D-10), commonly used to measure depression among older respondents [15, 35]. This scale includes negative-mood items, such as “I am troubled by some small things” and “I feel lonely”; items related to positive emotions, such as “I am happy” and “I am hopeful for the future”; and items related to somatic syndromes, such as “My sleep is not good.” Responses are assessed on a five-point Likert-type scale (5 = rarely or none of the time, 4 = not much of the time, 3 = almost half of the time, 2 = most of the time, and 1 = almost every day). Reverse questions are reverse coded. Summed scores represent the level of depressive symptoms (range: 10–50), with higher scores indicating lower levels of depression. In this sample, the Cronbach’s alpha estimate for the CES-D-10 was 0.76.

Life satisfaction was measured using 13 questions to enquire about the older adults’ satisfaction with their family relationships, financial situation, and living conditions. Responses are assessed on a five-point Likert-type scale (5 = very satisfied to 1 = very dissatisfied). Summed scores represent the level of life satisfaction (ranging from 13 to 65), with higher scores indicating higher levels of life satisfaction. The Cronbach’s alpha for this scale was 0.86 in this sample.

Loneliness was measured using the simplified UCLA Loneliness Scale (UCLA-6), which consists of three positive and three negative questions; responses are assessed on a three-point Likert-type scale (1 = yes to 3 = no). Reverse questions are reverse coded. Summed scores represent the level of loneliness (6–18), with higher scores indicating lower levels of loneliness. The Cronbach’s alpha for this scale was 0.61 in this sample.

### Independent variable

The independent variable was the intergenerational support from adult children. In this study, intergenerational support was assessed along four dimensions: instrumental, emotional, material, and financial support [13, 36]. Instrumental support was measured using the following item: “How much instrumental support has your child provided you in the past 12 months? (e.g., helping you with housework, cooking, and shopping).” Financial support was assessed using the item “How often have your children given you financial support in the past year?”. Material support was measured with the item “How often have your children given you material support (for example, buying food, cloths, and articles of daily use, rather than money) in the past year?”. Emotional support was assessed using the item “How often do your children communicate with you emotionally?”. Responses were collected using a four-point Likert scale, with responses ranging from 1 (almost never) to 4 (always). Summed scores represent the level of intergenerational support (range = 4–16; Cronbach’s alpha = 0.627), with higher scores indicating greater intergenerational support from adult children.

### Mediator variables

Older adults’ attitudes toward younger people and their willingness to interact with them in a non-familial context were used as mediator variables in this study. The older adults’ attitudes toward younger people were measured using the age group evaluation and description (AGED) scale, which includes four factors, goodness, positiveness, vitality and maturity [37]. Each factor comprises seven contrasting adjectival pairs (for example, friendly/unfriendly) arranged on a seven-point Likert scale. Summed scores represent older adults’ attitudes toward younger people within a non-familial context (range = 28–196), with higher scores indicating better evaluation. Cronbach’s alpha for this scale was 0.91.

The willingness to interact with younger people within a non-familial context was assessed through asking older adults whether they would like to participate with young people in relation to tea parties, joint trips, physical activities, learning or sharing history, and learning or practicing how to use electronic products. Responses were collected using a five-point Likert scale, with responses ranging from 1 (very reluctant) to 5 (very willing). Summed scores represent the willingness to interact with younger people within a non-familial context (range = 5–25), with higher scores indicating a greater inclination to interact. Cronbach’s alpha was 0.84.

### Covariates

We controlled for the respondents’ health status in the model by including further factors such as marital status, number of chronic diseases, self-rated health, and instrumental activities of daily living (IADL) difficulties. Marital status was coded as “with partner” = 1 and “without partner” (i.e., single, widowed, or divorced) = 0. Self-rated health was assessed using a five-point Likert scale. Respondents were asked whether they had any of the 14 most common chronic diseases among older adults in China. The answers were recorded as binary variables (0 = no, 1 = yes). Summed scores are used to represent the number of chronic diseases. The 7-item Barthel Index was used to measure IADL. The responses to this index were assessed using a three-point scale (0 = no difficulty at all; 1 = quite difficult, assistance needed; 2 = very difficult). The summed scores represent the respondents’ ability to complete IADL. The higher the score, the worse the participant’s ability to live independently. The Cronbach’s alpha for the scale in this study was 0.860.

### Statistical analysis

Categorical variables are presented as numbers or percentages, while continuous variables are presented as means and standard deviations (SD). Before establishing the structural equation model (SEM), the hypothesis of multivariate normality needs to be confirmed as a precondition using Mardia’s coefficient and confirmatory factor analysis (CFA) used to test the measurement model [35].

During this process, the internal consistency, reliability, and discriminating validity of the measurement models need to be assessed. As a measure of internal consistency reliability, the composite reliability (CR) value should be more than 0.70 [38], and acceptable at 0.6. Discriminant validity is evaluated via the square root of the average variance extracted (AVE) where the square root of the AVE of each latent construct needs to be higher than the inter-construct correlation [39, 40].

**Fig. 1:**
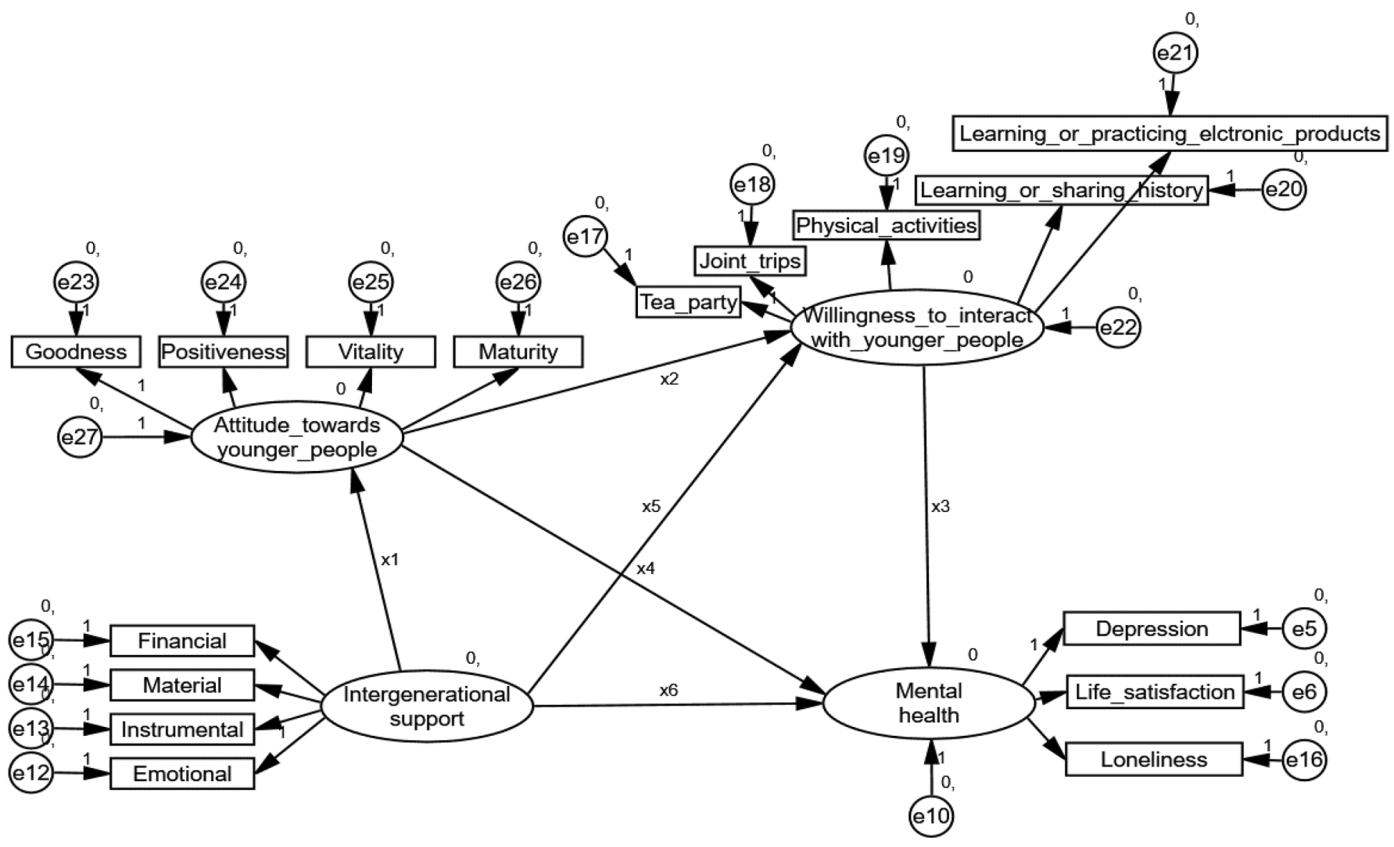
Hypothesized research model

Mediation is a way to explain the process or mechanism by which one variable affects another [41]. All mediation effects were examined using bootstrapping, estimating standard errors for indirect effects with 5,000 bootstrap samples, and providing 95% confidence intervals (CIs) [42, 43]. Statistically significant mediation effects can be inferred if the 95% CIs of the mean estimates do not include zero [44, 45]. SEM with two serial multiple mediation models was used to examine older adults’ attitudes toward younger people and their willingness to interact with younger people within a non-familial context, as serial mediators, in the relationship between intergenerational support and mental health (Fig. 1). A multiple serial mediation model with two mediators provides three specific indirect effects that sum to a total indirect effect. The specific indirect effects considered in this model were: (1) older adults’ attitudes toward younger people (X_1_*X_4_), (2) their willingness to interact with younger people within a non-familial context (X_5_*X_3_); and (3) older adults’ attitudes toward younger people and their willingness to interact with younger people within a non-familial context (X_1_*X_2_*X_3_). The final indirect effect comprised the specific indirect effects, which, if significant, would support serial multiple mediation [46]. Because AMOS does not output specific indirect effects, the syntax was rewritten so that AMOS outputs could be used to estimate specific indirect effects. Additionally, the percentage of the total effect explained by specific indirect pathways was calculated.

In addition, different goodness-of-fit indices were applied to confirm the adequacy of the SEM [47]: chi-square divided by degrees of freedom (χ^2^/DF), root mean square error of approximation (RMSEA), the incremental fit index (IFI), the comparative fit index (CFI), the normed fit index (NFI), and the Tucker-Lewis index (TLI). As recommended in previous literature, RMSEA and SRMR values smaller than 0.08, χ^2^/DF values smaller than 5, and IFI, CFI, NFI, and TLI values greater than 0.9 indicate an acceptable fit [48]. Data management and statistical analyses were performed using IBM SPSS Statistics software (version 25.0) and AMOS Version 20.0. Statistical significance was set at P values < 0.05.

## Results

### Descriptive analysis

The descriptive characteristics of the respondents are presented in Table 1. More than half of the respondents were female (61.4%; *n* = 479) and more than half were married (78.3%; *n* = 611). The average age of respondents was 70.47 years (SD = 7.05). Their mean IADL, number of diseases, and self-rated health scores were 0.58 (SD = 1.72), 1.63 (SD = 1.6) and 3.55 (SD = 0.82) respectively, indicating that they were in relatively good physical condition. The respondents’ average scores on the four dimensions of intergenerational support were 3 (SD = 0.97), 3.51 (SD = 0.7), 2.89 (SD = 1.02) and 3.53(SD = 0.75), respectively. On the AGED scale, the average scores on goodness, positiveness, vitality and maturity were 35.95 (SD = 6.94), 38.33 (SD = 6.18), 28.97 (SD = 6.36), 34.02 (SD = 7.4), respectively. The mean depression score was 36.77 (SD = 4.39), indicating a low level of depression. In addition, the respondents’ average loneliness and life satisfaction scores were 16.81 (SD = 1.79) and 53.59 (SD = 5.96), respectively. The mean score for willingness to interact with younger people within the non-familial context was 20.13 (SD = 3.98).

**Table 1:** Descriptive statistics of the sample (*N* = 780)

Variables	Mean	SD	N	%
*Gender*				
Male			301	38.60%
Female			479	61.40%
*Marital Status*				
Divorced or other			169	21.70%
Married			611	78.30%
Age	70.470	7.050		
IADL	0.580	1.720		
Number of diseases	1.630	1.600		
Self-rated Health	3.550	0.820		
*Intergenerational Support*				
Instrumental Support	3.000	0.970		
Material Support	3.510	0.700		
Financial Support	2.890	1.020		
Emotional Support	3.530	0.750		
*Attitudes toward Younger People*				
Goodness	35.950	6.940		
Positiveness	38.330	6.180		
Vitality	28.970	6.360		
Maturity	34.020	7.400		
*Mental Health*				
Depression	36.770	4.390		
Loneliness	16.810	1.790		
Life Satisfaction	53.590	5.960		
Willingness to Interact with Younger People	20.130	3.980		

Note: IADL = instrumental activities of daily living

### SEM reliability, validity, and presupposition testing

As shown in Table 2, all CR values were > 0.6, indicating a satisfactory level of internal consistency. Concerning the discriminant validity analysis, Table 3 shows that the square root of the AVE value of each construct was higher than its correlation with any other construct, indicating a high discriminant validity in the constructs. The discrimination of each factor in representing a different dimension was verified [49].

**Table 2:** Composite reliability

Construct	Variable	Factor Loading	Square Multiple Correlations (SMC)	Composite Reliability (CR)
Intergenerational Support	Emotional Support	0.490	0.240	0.640
	Instrumental Support	0.604	0.365	
	Material Support	0.610	0.372	
	Financial Support	0.513	0.263	
Attitudes toward Younger People	Goodness	0.886	0.785	0.909
	Positiveness	0.790	0.624	
	Vitality	0.816	0.666	
	Maturity	0.887	0.787	
Willingness to Interact with Younger People	Tea party	0.738	0.545	0.844
	Joint trips	0.783	0.613	
	Physical activities	0.801	0.642	
	Learning or sharing history	0.688	0.473	
	Learning or practicing with electronic products	0.585	0.342	
Mental Health	Depression	0.634	0.402	0.649
	Loneliness	0.595	0.354	
	Life Satisfaction	0.623	0.388	

**Table 3:** Discriminant validity

Average Variance Extracted (AVE)	Construct	Intergenerational Support	Attitudes toward Younger People	Willingness to Interact with Younger people	Mental Health
0.3100	Intergenerational Support	***0.557***			
0.7154	Attitude toward Younger People	0.235	***0.846***		
0.5230	Willingness to Interact with Younger People	0.004	0.238	***0.723***	
0.3814	Mental Health	0.119	0.316	0.257	***0.618***

Note: Diagonal italic values are the square roots of the AVE value of constructs

**Fig. 2:**
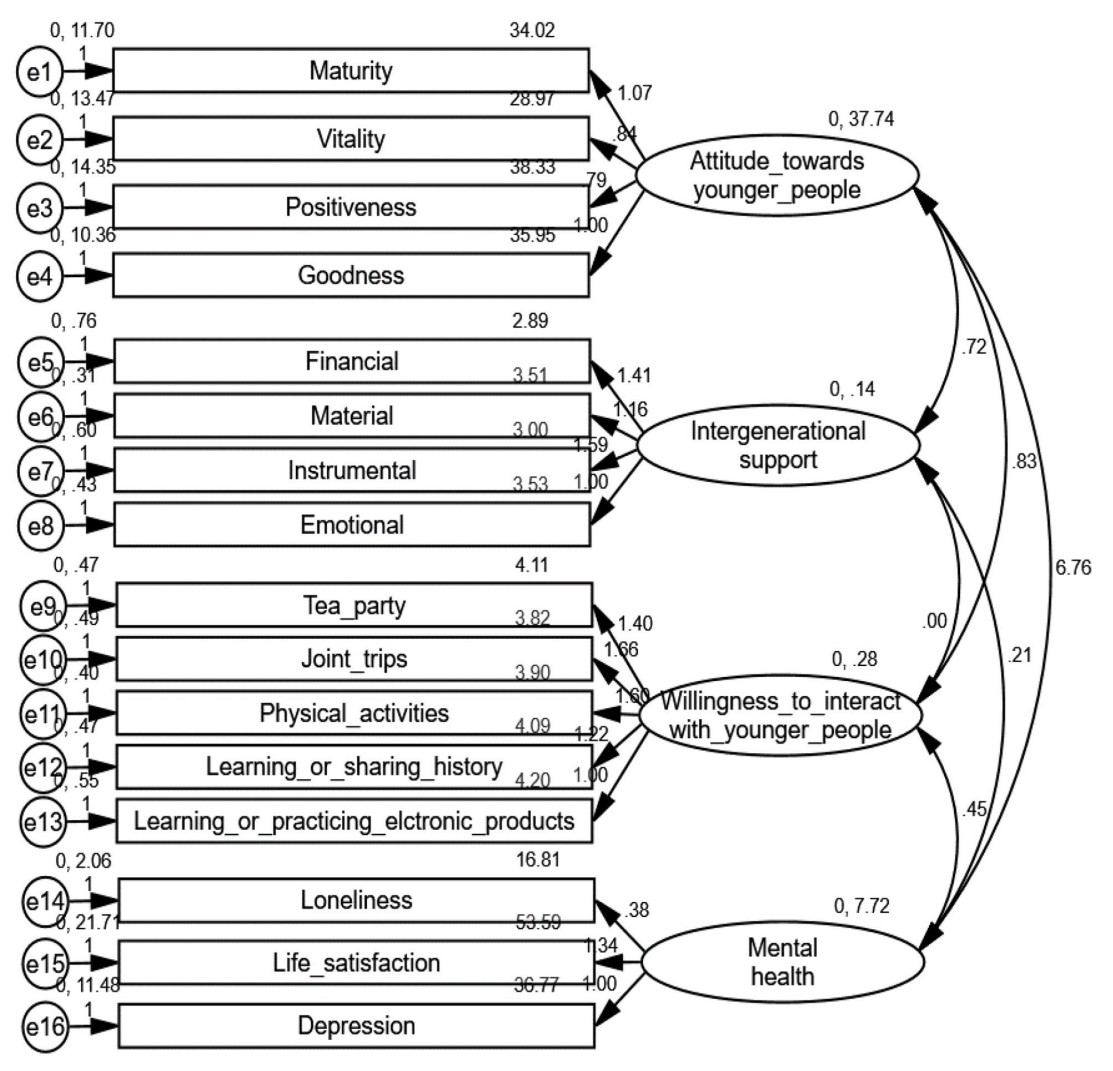
Confirmatory factor analysis (CFA)

Thus, the hypothesis of the multivariate normality of the data (Mardia = 58.348) was confirmed. This coefficient was less than 288, extracted from p *(*p* + 2), with “p” being the number of total variables in the scale (16) [50]. Furthermore, as shown in Fig. 2, the CFA showed a good model fit index: χ^2^ = 323.602, DF = 98, χ^2^ / DF = 3.302, NFI = 0.931, IFI = 0.951, TLI = 0.94, CFI = 0.951, RMSEA = 0.054.

### Hypothesis testing

**Fig. 3:**
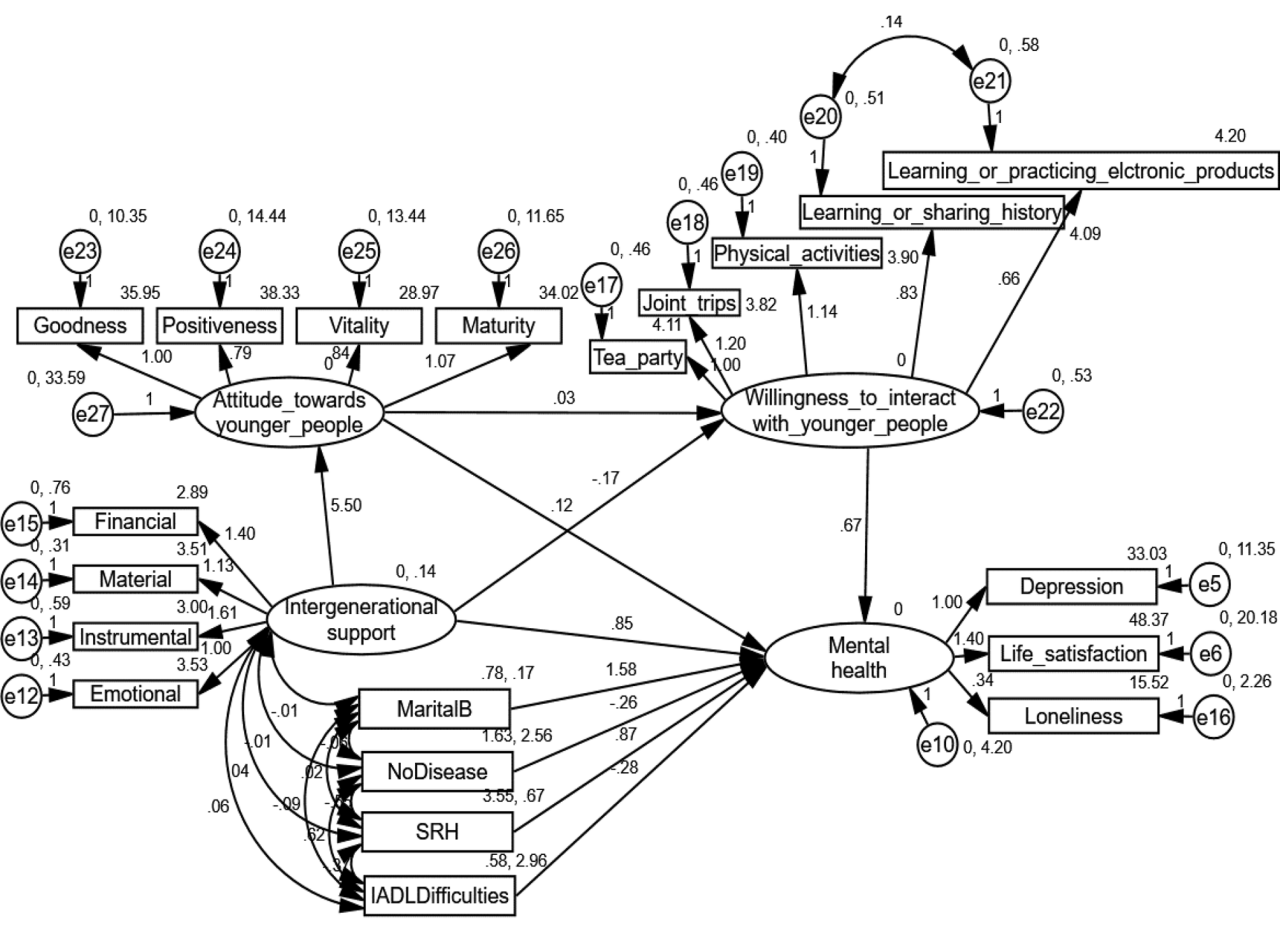
Final SEM model

Table 4 presents the path coefficients for the research models. Table 5 shows the indirect effects of intergenerational support on older adults’ mental health. The final SEM was obtained by increasing the residual correlation and modification indices, as shown in Fig. 3. The goodness-of-fit indices of the final model indicated an excellent fit (χ2 = 489.261, DF = 153, χ2 / DF = 3.198, NFI = 0.908, IFI = 0.935, TLI = 0.919, CFI = 0.935, RMSEA = 0.053). The prerequisite assumption of the SEM is that the residuals are independent and cannot be correlated unless there is a reasonable explanation. Residual correlation in the final model could be explained in terms of the item B224 “Willingness to share traditional history with younger people, such as paper cutting,” and the item B225 “Willingness to let younger people teach you how to use electronics,” as both items are about teaching each category of person certain skills; therefore, these items were considered to involve residual correlation.

**Table 4:** Parameter estimates from the structural equation model

Path	Label	β	B	SE	CR	P
Intergenerational Support → Attitudes toward Younger People	X1	0.332	5.503	0.871	6.318	***
Attitude toward Younger People → Willingness to Interact with Younger People	X2	0.279	0.034	0.006	6.184	***
Intergenerational Support → Willingness to Interact with Younger People	X5	-0.084	-0.17	0.106	-1.607	0.108
Intergenerational Support → Mental Health	X6	0.115	0.852	0.402	2.121	0.034
Willingness to Interact with Younger People→ Mental Health	X3	0.184	0.668	0.164	4.062	***
Attitudes toward Younger People → Mental Health	X4	0.271	0.121	0.021	5.701	***
Marital → Mental Health		0.238	1.578	0.268	5.877	***
No. of Disease → Mental Health		-0.153	-0.262	0.079	-3.31	***
SRH → Mental Health		0.259	0.87	0.154	5.633	***
IADL → Mental Health		-0.173	-0.275	0.074	-3.736	***

*Note: β* = Standardized regression coefficient; B = Unstandardized regression coefficient; SE = Standard error; CR = Critical value; SRH = self-rated health; IADL = instrumental activities of daily living, P-value for path analysis

**Table 5:** Direct effect, indirect effect and total effect

Specific Indirect Effect	Path	Bias Corrected percentile method (95% CI)			
		β	B	Lower	Upper
Int1	X1→X2 →X3	0.017	0.126	0.069	0.224
Int2	X1 →X4	0.090	0.665	0.443	1.046
Int3	X5 →X3	-0.015	-0.113	-0.267	0.011
Total Indirect Effect	Int1 + Int2 + Int3	0.092	0.677	0.407	1.075
Direct Effect	X6	0.115	0.852	0.157	1.617
Total Effect	Int1 + Int2 + Int3 + X6	0.207	1.530	0.858	2.331

*Notes: β* = Standardized regression coefficient; B = Unstandardized regression coefficient; CI = confidence interval

As shown in Table 4, all effects were significant, except for the effect of intergenerational support on willingness to interact with younger people within a non-familial context (*β* = -0.084, SE = 0.106, *P* =.108, P-value for path analysis). Specifically, intergenerational support from children had a significant positive direct effect on older adult mental health (*β* = 0.115, SE = 0.402, *P* =.034, P-value for path analysis), Hypothesis 1 was confirmed. Receiving support from children had a significant positive effect on older adults’ attitude toward younger people (*β* = 0.332, SE = 0.871, *P* <.001, P-value for path analysis). Attitudes toward younger people had a significant positive effect on willingness to interact with younger people within a non-familial context (*β* = 0.279, SE = 0.006, *P* <.001, P-value for path analysis); the better the attitudes toward younger people, the greater the tendency to interact with younger people within a non-familial context. Both the effects of willingness to interact with younger people within a non-familial context (*β* = 0.184, SE = 0.164, *P* <.001, P-value for path analysis) and attitudes toward younger people (*β* = 0.271, SE = 0.021, *P* <.001, P-value for path analysis) on older adults’ mental health were significant. The better the attitudes toward younger people and the greater the tendency to interact with younger people within a non-familial context, the better the mental health status of the older adults. All covariates had a significant effect on mental health; in particular, marital status (*β* = 0.238, SE = 0.268, *P* <.001, P-value for path analysis) and self-rated health (*β* = 0.259, SE = 0.154, *P* <.001, P-value for path analysis) had positive effects, whereas the number of diseases (*β* = -0.153, SE = 0.079, *P* <.001, P-value for path analysis) and IADL (*β* = -0.173, SE = 0.074, *P* <.001, P-value for path analysis) had negative effects.

The direct, indirect, and total effects are presented in Table 5; two of the three indirect effects were significant. Confirming Hypothesis 2, the specific indirect effect through older adults’ attitudes toward younger people was significant (X_1_*X_4_: B = 0.665, 95% CI [0.44, 1.046]) but the specific indirect effect through willingness to interact with younger people within a non-familial context (X_5_*X_3_: B = -0.015, 95% CI [-0.27, 0.011]) was nonsignificant. Finally, a significant indirect effect of intergenerational support on mental health through both attitudes toward younger people and willingness to interact with younger people within a non-familial context (X_1_*X_2_*X_3_: B = 0.126, 95% CI [0.07, 0.224]) was found, confirming Hypothesis 4. The association between intergenerational support and mental distress was statistically significant (total effect: B = 1.53, 95% CI [0.86, 2.331]), explaining 43.9% of the variance. In addition, the direct effect of intergenerational support on mental distress was statistically significant (direct effect: B = 0.852, 95% CI [0.16, 1.617]), indicating that this relationship was partially mediated by the mediators, with the indirect effect accounting for 44.44% of the total effect.

## Discussion

This study explored the complex relationship between intergenerational support and the mental health of older adults in urban China. Using the proposed structural model, we systematically examined the mediating roles of older adults’ attitudes toward younger generations and their willingness to interact with them in a non-familial context. A significant positive association was found between intergenerational support and the mental health of older adults in urban China (B = 0.852, 95% CI [0.157,1.617]). Furthermore, intergenerational support had a specific indirect effect on mental health through older adults’ attitudes toward younger people within a non-familial context (B = 0.665, 95% CI [0.443,1.046]). In particular, there was a chain mediation effect (B = 0.126, 95% CI [0.069,0.224]) in relation to attitudes toward younger people and the willingness to interact with younger people between intergenerational support and mental health. Mediation accounted for 44.44% of the total effects in the model. Our findings shed light on the multifaceted dynamics of intergenerational support and its implications for geriatric mental health and offer valuable insights for policymakers and researchers. Consistent with Hypothesis 1, we observed a positive effect of intergenerational support on the mental health of older adults. This observation aligns with empirical findings showing a marked enhancement of geriatric mental health with increased support from children [10–13, 15, 51]. In Chinese culture, intergenerational support is considered one of the most important sources of support for older adults. The ethos of filial piety, which is deeply embedded in traditional Chinese culture, underscores the significance of filial action in sustaining intergenerational relationships [52]. Consequently, receiving intergenerational support could be crucial in meeting older parents’ social expectations and bolstering their sense of security, especially in the face of financial difficulties, health challenges, and psychological distress in their daily lives [11]. Notably, our conception of intergenerational support helps clarify the effects of intergenerational support on mental health, as inconsistent findings on this relationship have been reported in previous research.

We also identified a positive effect of intergenerational support on attitudes toward younger people. This result accords with understandings in affective transfer theory [28–29] and is further supported by empirical studies emphasizing how negative attitudes toward other age groups may be rooted in peer relationships within an age-segregation context. Conversely, ample intergenerational interactions and information exchange within the family context are pivotal in nurturing positive images in relation to age [27]. It is noteworthy that intergenerational support was not found to affect the willingness to interact with younger people within a non-familial context; a plausible explanation may lie in the adaptive strategy applied during the process of aging. According to continuity theory, older adults experience withdrawal from social activities. As an adaptive strategy, they are more likely to use available resources to preserve their sense of stability and maintain their social roles [53]. This non-significant outcome potentially underscores the preference of older Chinese adults to prioritize familial intergenerational support as the primary mechanism for navigating the aging process.

Our findings also showed a significant relationship between older adults’ attitudes toward younger people and their willingness to interact with them in a non-familial context. The more positive the attitudes toward younger people, the more likely the older adults were to interact with them. This finding accords with previous studies on the effectiveness of intergenerational programs in showing that fostering positive attitudes toward other age groups could be the first step in promoting intergenerational solidarity at the community level [54]. Both the effects of attitudes toward younger people and willingness to interact with younger people within the non-familial context on mental health were significantly positive, implying that combating age-related prejudice and achieving stable intergenerational ties are protective factors for the mental health of older adults and realizing age-friendly environments to promote healthy and active aging.

Drawing from the insights derived from our proposed serial multiple mediation model, the intricate relationship between intergenerational support and geriatric mental health can be partially elucidated through the mediating mechanism of attitudes toward younger people and the willingness to interact with them in the non-familial context. Specifically, older adults who received more intergenerational support from their adult children tended to have more positive attitudes toward younger people. These positive attitudes subsequently affected their willingness to interact with younger people outside the familial context, thereby mitigating potential mental health challenges. In contrast to the relevant literature that emphasizes intergenerational support with family members when examining protective factors for the mental health of older individuals [9–13], our findings indicate that the characteristics of intergenerational interaction at the micro level influence the macro elements of intergenerational solidarity, which in turn affect mental status at the individual level. This suggests that the understanding of intergenerational support needs to be expanded and refined when seeking to identify modifiable factors that can affect mental health among older adults, given the trends of urbanization and family nucleation. Moreover, the approach adopted in this study revealed the mechanism involved in forming attitudes that predict social interaction behaviors within a non-familial context. Studies on combating negative attitudes and behaviors between older and younger generations have not examined the specific conditions in which attitudes can be predictive of behaviors with regard to achieving intergenerational solidarity at the community level [55, 56]. The chain mediation model presented in this study facilitates greater understanding of this phenomenon: attitudes toward younger people among older adults are based on the direct experience of intergenerational interaction within the family, which in turn stimulates greater attitude-behavior consistency in terms of building harmonious ties with younger generations in society.

### Implication and limitation

The findings of this study have both practical and policy implications. First, this study highlighted the crucial role of intergenerational support provided by adult children for older adults, which not only has a direct protective effect on the mental health of older adults but also reduces their prejudice against younger people. Policymakers and other practitioners need to promote mental health among older adults by providing opportunities and social services to nurture family obligations and social exchanges across generations. Second, as the serial multiple mediation model showed, social policies and programs to achieve intergenerational solidarity in age-friendly communities need to be aware of the synergistic effects of intergenerational support at the family level and intergenerational interaction at the community level. Involving family members in the service delivery process is a commendable strategy for social workers and volunteers. Finally, we would contend that the understanding of intergenerational support needs to be expanded and refined given the geographical dispersal and nucleation increasingly affecting the family structure, which has otherwise weakened the intergenerational support needed for the long-term care of older adults. Consequently, researchers and policymakers need to consider intergenerational interactions and solidarity from a more macro perspective. It is plausible that the framework of intergenerational relationships and solidarity at the community or societal levels may emerge as even more significant than that at the familial level in terms of closeness and significance.

Our study has some limitations. First, quota sampling was employed to recruit respondents, implying that the sample was not procured through random selection. Thus, the findings should be approached with caution in that they are best understood as being provisional and representative, with primary reference to older adults in urban Chinese communities that share similar sociocultural characteristics. Second, the cross-sectional nature of our research design precluded the determination of causality, although our indirect effects were consistent with the mediation effects. Finally, there is the possibility of recall bias stemming from the self-reporting procedure, which may have affected the validity of the results.

## Conclusions

Through constructing a serial multiple mediation model, this study explored how intergenerational support is associated with mental health and the potential mediating mechanisms among older adults in urban China. We found that intergenerational support was positively associated with mental health status. Additionally, attitudes toward younger people and the willingness to interact with younger people within a non-familial context played important roles in mediating the relationship between intergenerational support and mental health. These findings are likely to be useful in helping to identify modifiable factors that can improve the mental health of older adults. This study’s findings enhance practical awareness of the synergistic effect of intergenerational support at the family level and intergenerational interaction at the community level.

## Data Availability

Data in this paper are available upon reasonable request to the corresponding author.
